# Supplementary material for: Are carbon emissions trading and green financial instruments synergistic? -Comprehensive quantitative research based on content analysis
Source: PLoS One. 2024 Mar 7;19(3):e0298601. doi: 10.1371/journal.pone.0298601 (PMC10919601; doi:10.1371/journal.pone.0298601)
Supplement: S1 Data — (ZIP) [file pone.0298601.s001.zip › Data/地级市Z_F工作报告.zip!/║╙─╧/σo╤⌠╩╨/└┤╫╘╣1⁄2╓┌║┼í╛┬φ┐╦╩2╛▌═°í┐_2004.docx]

各位代表：

现在，我代表市人民政府，向大会作政府工作报告，请予审议，并请各位政协委员提出意见。

一、过去五年工作的回顾

1999年以来，本届政府在省委、省政府和市委的领导下，高举邓小平理论伟大旗帜，全面贯彻“三个代表”重要思想，团结带领全市人民努力奋斗，战胜了非典疫情、严重旱涝等各种自然灾害，顺利地完成了市四届人大一次会议确定的各项任务，使我市呈现出经济发展、社会进步、大局稳定的良好局面。

国民经济快速发展。胜利完成“九五”计划，“十五”计划正在顺利实施。全市生产总值由1998年的170.6亿元增加到2003年的265.3亿元，增长60%，年均增长9.9％；人均生产总值由5032元增加到7502元，增长44.4%，年均增长7.6%。三次产业比例由1998年的23.6:51.8:24.6调整为2003年的16.1:59.5:24.4。

“以工兴市”成效显著。2003年全市工业增加值完成129.9亿元，比1998年增长70.9%，年均增长11.3%；限额以上工业增加值完成103.1亿元，首次突破百亿元大关。地方工业增加值完成59.8亿元，增长127.8%，年均增长17.9%。五年累计新上200万元以上的工业投资项目545个，总投资96亿元，工业产值和工业利税分别增加140亿元和16亿元。全市人民关注的林纸一体化一期工程和2×20万千瓦热电机组项目开工建设。三强集团、濮耐公司、光璞石化、力诺玻璃等企业的一大批工业项目建成投产。中原油田、中原乙烯、中原大化三大支柱企业加快发展。全市"双超"企业由1998年的5家增加到19家。

农村经济稳步增长。2003年全市农业增加值42.75亿元，比1998年增长34%，年均增长6%。濮阳县、清丰县、南乐县、范县、台前县、华龙区和高新区的农副产品、畜牧养殖和加工业稳步发展，产业化水平不断提升。26户重点龙头企业的产业化带动能力不断增强。全市优质粮食作物种植面积达到283万亩，占总面积的56%；优质蔬菜、名优花卉产量增加，效益提高。生态建设成效显著，全市退耕还林面积23.2万亩，农田林网控制率达到90%，森林覆盖率达到22%，被国家授予“全国绿化先进集体”称号。农田水利基本建设进一步加强，5年新增有效灌溉面积15.9万亩、旱涝保收田18.7万亩、节水灌溉面积49.5万亩。金堤河一期治理、引黄入鲁和第三濮清南干渠等工程建设全面完成，连续四年荣获全省市级“红旗渠精神杯”。扶贫开发工作取得新成绩，累计有16.5万人脱贫。

重点项目建设实现突破。5年累计完成全社会固定资产投资395.7亿元。特别是重点项目实现了新突破：一是交通基础设施建设迈出新步伐。濮鹤高速公路累计完成投资10亿元，路基、桥涵等工程完工；汤台铁路改造工程累计完成投资1.7亿元，完成了总投资的50%；濮信高速公路濮阳段开工建设，连接德商高速公路的濮范段正在做前期准备工作。二是筹建了一批大型地方工业项目。投资15.5亿元的林纸一体化项目和投资17.1亿元的2×20万千瓦热电机组项目已经开工建设，这是建市以来地方投资最大的工业项目，为我市的发展增强了后劲。三是建设了濮阳职业技术学院，新校区完成投资1.26亿元，已有3000名师生入住，结束了濮阳没有大学的历史。四是每年确定的重点项目都超额完成计划目标，每年承诺为市民办的实事都全面完成。

非公有制经济和第三产业迅速崛起。2003年全市非公有制企业达到7268家，个体工商户5.2万户，从业人员43.3万人；完成增加值109.2亿元，占全市生产总值的41.2%，年均递增19.3%。以民营投资为主体，商贸流通全面推进，2003年全市社会消费品零售总额达到73.3亿元，比1998年增长62.2％，年均增长10.2%。旅游业快速发展，绿色庄园被评为4A级景区，世锦园和毛楼生态区被评为3A级景区，濮上园、濮外园建成开园。

财政金融稳定运行。2003年全市财政收入11.3亿元，比1998年增长56.2%，年均增长9.3%；财政支出23.6亿元，比1998年增长101.4%，年均增长15%。财政保障能力明显增强，保证了干部教师工资按时足额发放和各项重点支出的需要。金融机构各项存款余额达到251.1亿元，比1998年增加97.9亿元；各项贷款余额达到138.2亿元，比1998年增加58.6亿元。

城镇化建设跃上新台阶。拉大了城市框架，完善了城市功能，巩固了“四城”创建成果，提升了城市品位。5年新修和改造城市道路26条，长35.2公里。市体育场、污水处理厂建成投入使用。第二水源供水工程建设，城内马颊河、濮水河段治理和创建生态市、文明城市等活动，使城市环境进一步优化。荣获了2001年中国首届人居环境范例奖、2002年迪拜国际改善人居环境良好范例奖、2003年“国际花园城市”金奖，“五城二奖”使我市在中西部地区独树一帜。5个县突出县域特色，全面加快小城镇建设，取得了可喜成绩。2003年，全市城镇化率达到25.6%。

改革开放不断扩大。国有企业改革不断深化。全市118户国有工业企业改制面达到78.8%，盘活有效资产6亿多元，安置职工5300人，吸纳市外资金9700多万元。医疗保险制度改革不断深化。完成了市县乡党政机构改革。积极推进了行政审批制度改革，建立了市行政服务中心及10家分中心、19家工作站，审批项目由2002年的2128项压减到353项。完成了农村税费改革，亩均减负53.4%，人均减负53.3%。财税、金融、流通和住房制度改革等稳步推进。整顿和规范了市场经济秩序，进一步优化了经济发展环境。2003年，全市引进外资实际到位3021万美元，比1998年增长1.6倍；出口创汇6760万美元，增长4.3倍。市高新区和工贸示范区5年内新上百万元以上项目869个，引进到位资金86.7亿元。

社会事业全面进步。大力实施“科教兴濮”战略。5年来，实施国家科技型中小企业技术创新项目3项、国家和省火炬项目30项、省高新技术产业化重点项目12项，培育国家和省高新技术企业23家，开发国家和省级高新技术产品62种。教育事业不断进步，有82个乡（镇）达到“普九”提高标准，占全市乡（镇）总数的96.5%。全市普通高考省划上线率和重点大学录取人数连续7年位居全省前列，被清华、北大、人大、科技大四所名校录取人数连续5年居全省首位。人口和计划生育工作取得新成绩，人口自然增长率年均低于全省平均水平。文化事业全面发展，豫剧《村官李天成》等3个节目获省“五个一工程奖”，东北庄被命名为“全国杂技之乡”，杂技节目多次荣获国际、国内大奖。成功举办了四届龙文化活动周和建市20周年庆典活动。去年面对突发的非典疫情，全市上下万众一心，众志成城，全力抗击非典，取得了重大胜利，实现了“非典”为零的目标，守住了河南的“北大门”。爱国卫生运动和全民健身活动深入开展，荣获“全国控制吸烟先进城市”称号。城乡医疗设施和卫生条件不断改善，并实施了省新型农村合作医疗试点县工作，市人民医院等4家医院被授予“全国百姓放心医院”。在安全生

产方面，强化了安全生产责任制，近3年未发生重大安全事故。加强土地资产管理，土地收益大幅度增加。社会保障体系建设进一步完善，国有企业下岗职工基本生活费和企业离退休人员养老金按时足额发放，城市“低保”应保尽保。就业再就业工作进一步扩大，5年累计安置国有企业下岗职工4.5万人，其中2003年安置下岗失业人员再就业20372人，安置“4050”人员5282人。人民生活进一步改善，2003年全市城镇居民人均可支配收入达到6327元，比1998年增长35.5%，年均增长6.3%；农民人均纯收入达到1948元，增长18.5%，年均增长3.4%。环境保护、物价、审计、统计、广播电视、民兵预备役、民族宗教、外事侨务、史志、档案、地震、气象、人防、残疾人事业等都取得了新的成绩。

精神文明和民主法制建设进一步加强。“三个代表”学习教育活动卓有成效。落实公民道德建设实施纲要，广泛开展了机关效能建设，使广大干部群众精神风貌为之一新。严格执行人大及其常委会的决定、决议，自觉接受人大法律监督和政协民主监督，认真办理人大代表议案、建议和政协提案。支持各民主党派、工商联参政议政。加强社会治安综合治理，严打整治斗争取得新成果。同“法轮功”等邪教组织的斗争取得重大胜利。廉政建设和反腐败斗争取得新成效。密切党群干群关系，重视人民来信来访，妥善解决群众反映的热点、难点问题，社会大局保持稳定。

过去五年取得了很大的成绩，主要是我们坚持了以经济建设为中心，聚精会神搞建设，一心一意谋发展；坚持了全心全意为人民服务的宗旨，立党为公，执政为民；坚持了解放思想，转变观念，抓住机遇，把握大局，调动一切积极因素，实现跨越式发展；坚持了改革开放，维护社会稳定，为全面建设小康社会提供强大动力和坚实保障；坚持了“科教兴濮”和可持续发展战略，促进经济社会发展与人口资源环境相协调统一；坚持了大力发展先进文化，推动物质文明与精神文明协调发展。

各位代表，过去五年的成绩，是省委、省政府和市委正确领导的结果，是全市人民共同奋斗的结果。在此，我代表市人民政府向全市广大工人、农民、知识分子、干部、人民解放军和武警官兵以及各界人士致以崇高的敬意，表示衷心的感谢!

在肯定成绩的同时，我们必须清醒地看到，我市经济社会发展中还存在一些困难和问题。主要是城乡居民收入特别是农民收入水平偏低，部分群众生活还很困难；地方工业总量仍然偏少；城镇化进程滞后；产业结构不合理，二、三产业比重还比较小，非公有制经济发展不足；金融机构存贷反差较大；社会保障和再就业任务繁重；县乡财政仍较困难；市场经济秩序有待继续整顿和规范。对此，我们一定高度重视，在今后的工作中着力予以解决。

二、今后五年工作的总体要求、奋斗目标和主要任务

今后五年是全面建设小康社会的重要时期。政府工作的总体要求是：以邓小平理论和“三个代表”重要思想为指导，全面贯彻党的十六大和十六届三中全会精神，树立科学的发展观；坚持“五个统筹”，围绕全面建设小康社会，以结构调整为主线，以改革开放和科技进步为动力，以提高人民生活水平为根本目的，深入实施“以工兴市”战略、科教兴濮战略、开放带动战略、可持续发展战略，加快工业化、城镇化和农业现代化进程，促进国民经济持续快速协调发展，实现速度和结构、质量和效益相统一；正确处理改革、发展、稳定的关系，推动社会全面进步，促进社会主义物质文明、政治文明、精神文明协调发展。

今后五年经济和社会发展的主要预期目标是：全市生产总值年均增长10％；全社会固定资产投资年均增长12％；社会消费品零售总额年均增长10％；外贸出口年均增长12％，实际利用外资年均增长20％；地方财政收入年均增长10％；城镇居民人均可支配收入年均增长6％，农民人均纯收入年均增长5％；年人口自然增长率控制在7‰以内；城镇化率达31％；每年新增城镇就业岗位3.2万个，城镇登记失业率控制在4.5％以内。

为实现上述目标，要突出抓好6个方面的工作：

第一，坚持“以工兴市”，加快工业化进程。大力实施“以工兴市”战略，加快经济发展。要始终坚持结构调整和体制创新，深化国有企业改革，大力发展开放型经济和非公有制经济，建设服务型政府。坚持以市场为导向，优化工业结构，形成以高新技术产业为先导、基础产业和制造业为支撑、服务业全面发展的格局。培育和扶持新的经济增长点，走新型工业化道路。要深化改革，推进创新，让一切劳动、知识、技术、管理和资本的活力竞相迸发，让一切创造社会财富的源泉充分涌流，为濮阳发展注入新的动力和活力。

第二，坚持投资拉动，狠抓重点项目建设。为加速经济发展，必须坚持“以工兴市，项目支撑”的方针，扩大经济增量，增加固定资产投入。要紧紧抓住国家政策性投资向重大项目倾斜的机遇，积极筛选、申报项目，争取国家和省的资金支持。要加大投融资体制改革力度，拓宽外资、民资和社会资金的融资渠道，引导更多的资金进入经济建设领域。要坚持以项目建设为载体，争取更多的重大基础设施、城市建设、交通、工业、农业、商贸旅游服务业等重点项目落户我市。

第三，坚持城乡统筹，发展壮大县域经济。要把县域经济作为统筹城乡经济协调发展的重要结合点，摆在突出位置来抓。充分发挥油气资源、农副产品资源优势，千方百计上一批大型、特大型工业项目和农副产品加工项目，加快工业发展步伐。进一步解放思想，更新观念，放手发展非公有制经济，带动县域经济的新跨越。扩大对外开放，引进外来资金、技术、人才和先进管理经验，为县域经济发展注入活力。搞好小城镇建设，充分发挥小城镇的聚集作用和载体功能，打造县域经济发展新平台。加大投入，加强农业基础设施建设，加快发展现代农业，着力解决“三农”问题，奠定县域经济稳定发展的基础。

第四，坚持“科教兴濮”，实施“人才强市”。深化科技体制改革，增加科技投入，加快创新体系建设，加强科技推广与普及，提高科技进步对经济增长的贡献率。扎实推进基础教育，加快职业教育、成人教育、高等教育发展步伐，规范和发展民办教育，尽快建立适应我市经济社会发展需要的复合型人才培养体系；加大教育特别是农村教育的投入力度，改善城乡办学条件。创新人才工作机制，完善和落实人才政策，加大高层次人才的培养和引进力度，建立优秀人才脱颖而出和人尽其才的有效机制和良好环境。

第五，坚持统筹兼顾，发展各项社会事业。坚持科学发展观，推动经济社会协调发展，促进人与自然和谐相处。增加促进社会发展和保护资源、环境的投入。加强公共卫生体系建设，全面提升卫生服务能力和整体工作水平；加快农村卫生事业发展，改善农民卫生服务条件；深化城镇医疗卫生体制改革，不断增强疾病防控能力。深化文化体制改革，加强文化基础设施建设，促进文化事业和文化产业协调发展。深入开展全民健身活动，增强全民体质。加强人口与计划生育工作，保持低生育水平，提高人口素质。依法保护、合理开发资源，做好污染防治工作。

第六，坚持执政为民，改善人民群众生产生活条件。始终坚持立党为公，执政为民，切实维护广大人民群众的根本利益。把扩大就业再就业摆上突出位置，积极培育和拓展新的就业岗位，拓宽就业领域，广开就业门路。依法加强劳动用工管理，维护广大职工和农民工的合法权益。建立健全社会保障体系，完善城镇职工基本养老保险、基本医疗保险制度，扩大各项社会保险覆盖面。加大扶贫攻坚力度，探索扶贫开发新机制，改善贫困地区农民生产生活条件，使更多的贫困农民脱贫致富。市政府每年都要列出与人民群众生活息息相关的实事，公开向全市人民承诺，保证落实兑现，使广大群众在改革发展中获得更多的实惠。

三、2004年的重点工作

今年是新一届政府的开局之年。做好今年的政府工作，开好头，起好步，意义重大。我们一定要认清形势，统一思想，把我市放在全国和全省发展的大背景下审视自己，总结经验，找准位置，找准差距，采取超常措施，奋起直追，为全面建设小康社会和中原崛起积极工作。这不仅是我们的职责，也是我们的历史责任。对此，我们不能有丝毫的满足和懈怠。必须坚持发展不动摇，一心一意搞建设，全心全意搞发展，牢牢坚持“五个统筹”，紧紧围绕加快“三化”进程，狠抓以工兴市和县域经济，重点突破，整体推进，跳跃式发展，打造经济强市。全市各行各业在工作中要创业绩、争一流，各方面工作在全省晋升位次，为濮阳市的小康社会建设作贡献。

2004年经济和社会发展的主要预期目标是：全市生产总值增长10%以上，全社会固定资产投资增长12%，地方财政收入增长11%，社会消费品零售总额增长10%，外贸出口增长12%，实际利用外商直接投资增长20%，城镇居民人均可支配收入增长8%，农民人均纯收入增长5%，城镇新增就业岗位3.2万个，下岗失业人员再就业1.72万人，城镇登记失业率控制在4.3%以内，人口自然增长率控制在5.6‰以内。工业企业经济效益综合指数、实现利润质量和效益指标要进一步提高。今年，重点抓好十个方面的工作：

（一）大力实施“以工兴市”战略，加快工业化进程

实现新型工业化，是我市现代化进程中艰巨的历史性任务。实施“以工兴市”战略，是实现我市工业化的重要途径和措施，我们必须贯彻始终，全面推进。一要做强做大石化主导产业。要创造良好环境，支持中原油田、中原乙烯、中原大化加快发展。加强油地合作，围绕油气开发和初级石化产品的利用，大力发展接续产业和精细化工，全面建设石化产业体系。二要着力培育地方大型企业集团。支持三强、力诺、濮耐、甲醇、贝利等重点工业企业通过资本运营等手段，吸纳社会资本，壮大实力，向大型企业集团迈进。三要加强工业区建设。重点扶持濮西、濮东、文留、柳屯、濮城、马庄桥、孙口、元村等8大工业区，使其成为推动濮阳工业发展的增长点。四要搞好食品加工、耐火材料、生物制药、电子信息等高新技术产品的生产。积极支持和鼓励民营企业开发高新科技工业项目。五要集中力量抓好工业重点项目建设。着重抓好林纸一体化项目、2×20万千瓦热电机组等项目的建设进度；市三胺化工1.5万吨三聚氰胺项目和濮阳县垃圾发

电项目年内要建成投产；县城电网改造、清丰220千伏输变电工程等电力项目年内要开工建设；全力支持中原油田油气勘探开发工程、中原乙烯5万吨混合碳四综合利用和中原大化3万吨三聚氰胺项目建设；中原大化30万吨甲醇项目争取年底动工；积极做好盐化工项目的前期准备工作。

（二）富民强县，推动县域经济大发展

加快县域经济发展，是打破城乡二元结构、实现城乡一体化发展的必然选择。县域经济发展，要坚持以工业化为主导，以城镇化为支撑，以农业现代化为基础，主攻民营经济，培育特色经济，壮大集群经济，发展配套经济，提升劳务经济。主攻民营经济，就是把发展民营经济作为主攻方向，思想上再放胆，准入上再放开，融资上再放活，政策上再放宽，优化环境，招商引资，示范带动，政策扶持，支持中小企业、大型企业竞相发展。培育特色经济，就是在你无我有，你有我优，你优我特上下功夫，注重发挥比较优势，宜农则农，宜工则工，宜商则商，宜服务业则服务业。壮大集群经济，就是发挥劳动力、农产品、油气资源丰富的优势，大力发展羽绒、玻璃、塑编、面粉等加工业，培育支柱产业，形成规模效应。发展配套经济，就是县域经济要主动服务于城市，大力发展城市生产、生活所需要的绿色食品、工业原料和服务业；积极加入大企业集团、优势企业的产业分工协作体系，搞好配套加工。提升劳务经济，就是要加强劳务培训，满足市场对较高层次劳务输出的需求，多层次、多渠道向国内外输出劳务，精心打造劳务输出品牌。全市都要加大对县域经济的扶持力度，各种政策要向县里倾斜，让县域经济成为我市经济发展的基石。

（三）以增加农民收入为核心，做好农业和农村工作

要把解决好农业、农村和农民问题，作为各项工作的重中之重，创新体制，落实政策，增加投入，用工业化理念发展农业，增加农民收入。一是大力培植农业产业化的龙头，促进农产品的加工增值转化。26户重点龙头企业要加快发展，增强辐射带动能力。加强农产品批发市场建设，积极发展农村经济合作组织和中介服务组织。二是搞好结构调整，加强基地建设。要积极推进农业和农村经济结构战略调整，大力发展优质、高产、高效、生态、安全农业，建立健全农业质量标准体系和农产品检验检测体系，提高农产品质量和竞争力。濮阳、清丰等县区要建设成国家优质小麦基地，黄河滩区和城区近郊要加快绿色奶业示范带和奶牛养殖基地发展。要搞好林业生产和生态环境建设，运用退耕还林政策、加快通道绿化和农田林网建设。各县（区）要按照与市签订的责任目标，落实责任，明确任务，严格考核，建设造纸速生丰产林基地。三是确保粮食安全。在确保耕地保护面积的基础上，落实国家促进粮食生产的各项政策，调动农民种粮积极性，依靠科技进步，改进生产方式，稳定和提高粮食生产能力。四是拓宽农民增加收入的渠道。大力发展农村二、三产业，壮大农村民营经济等非公有制经济；加强对农村劳动力职业技能培训，做好外出劳务人员的组织、协调和管理工作，引导和支持农民外出务工，多渠道增加农民收入。五是按照上级统一部署，深化农村税费改革，减轻农民负担。六是加强农村基础设施建设。搞好灾后重建和水毁工程修复，加快渠村灌区和南小堤灌区配套建设，完善黄河防洪工程，狠抓金堤河内涝治理，完成范县陆集移民建镇村台主体淤筑。建设好国家支持的人畜饮水、乡村道路、农村沼气、节水灌溉等小型工程。完善兴农网，加快农业信息化进程。搞好扶贫开发和以工代赈工作，减少和防止返贫。

（四）进一步放宽放活政策，大力发展非公有制经济

非公有制经济是最具潜力和活力的经济增长点，要坚定不移地把非公有制经济作为经济工作的战略重点来抓。一是落实政策措施。放宽非公有制经济的市场准入条件，营造有利于非公有制经济发展的良好环境，鼓励非公有制经济参与文化、教育、卫生、旅游等事业发展，支持民间资本投入基础设施和公用设施的建设与经营，放手、放开、放胆发展非公有制经济。二是完善金融服务，认真解决民营企业融资难问题。国有商业银行要制定相应的支持政策，加大信贷投入；城市信用社、农村信用社要把民营企业作为主要服务对象，新增贷款要向他们倾斜。三是坚持内外并举，激活民资和引进外资一起上。充分发挥市高新区、市工贸示范区的示范带动作用，吸引非公有制企业进区入园发展，形成新的聚集效应。大力引进外部资金、人才和资源，增加发展的新动力。

（五）推进各项改革，扩大对外开放

按照中央和省的部署，有重点有步骤地推进改革。一是以产权制度改革为重点，深化国有企业改革。因企制宜，一企一策，突出搞好16户国有企业改革工作，林州钢铁公司、中原啤酒厂等7户企业完成股份制改造，市一机厂等9户企业完成破产清算。规范法人治理结构。分离企业办社会职能。二是巩固行政审批制度改革成果，加强行政审批监管。凡取消或下放的项目，有关部门不得变相审批和收取费用。对保留的审批事项，要规范程序，简化手续，做到及时高效。同时按省要求，完成事业单位人事制度改革任务。三是加快国有粮食购销企业改革，切实解决粮食企业的“老人、老粮、老账”等问题。今年要从粮食风险基金中拿出部分资金，对种粮农民实行直接补贴。四是推进投资体制改革，建立“谁投资、谁决策、谁收益、谁承担风险”的投资机制，使企业真正成为投资主体。加强对政府投资的监督管理，提高政府投资效益。

大力实施开放带动战略。抓住国内外特别是沿海地区产业转移的战略机遇，以项目为载体，以优化环境为基础，大力引进境外和市外资金、技术、管理及人才。特别要突出引进规模大、技术含量高、带动能力强的大型企业集团。要搞好优质服务和环境建设，使引进来的项目留得住，发展快，实现互赢互利。积极参加第三届河南省投资贸易洽谈会，拓宽外联渠道。落实外贸出口鼓励政策，推动外贸经营主体多元化，发展特色出口商品，形成新的创汇优势。积极实施“走出去”战略，要搞好服务，简化手续和环节，鼓励和支持各类企业对外承包工程。

（六）坚持“三头并举”，加快城镇化步伐

按照“三头并举、突出重点、协调发展”的要求，加快城镇化进程。“三头并举”，就是城市、县城、小城镇全面推进，竞相发展。“突出重点”，就是在全面推进的基础上，实施重点突破，以城带乡，以市带县促镇。市区要巩固“五城二奖”创建成果，拉大框架，完善功能，提高品位，充分发挥对全市城镇化的辐射、带动作用。今年确定的15个城建重点项目，要采取措施，全面完成建设任务。5个县城建设要根据规划全面推进，扩大规模，加快发展。小城镇要在规划指导下，因地制宜，突出特色，注重实效。同时，要继续抓好120个“西辛庄式的小康示范村”的建设。“协调发展”，就是城镇经济社会要协调推进，成为生产发展、生活富裕、生态优美的新型社区。加强城市规划管理，维护城市规划的法律性和严肃性。要提高城市建筑设计水平，打造濮阳城市特色。加大经营城市力度，按照“谁投资、谁收益”的原则，推进投资行为社会化，筹资主体多元化，融资渠道多样化，引进外资和社会资金参与城镇建设和管理。进一步消除城镇化的体制性障碍，鼓励有条件的农民进城，促进人口、资金、产业向城镇聚集。城

镇要强化产业支撑，培育支柱产业和特色经济，做到产业支撑城镇建设，城镇搭建产业发展平台。

（七）加强重点项目建设，扩大投资消费需求

切实抓好关系濮阳发展全局的重点项目建设，扩大投资需求。今年要着力抓好产业和基础设施方面的142个重点项目，其中工业项目90个、农业项目4个、城建项目15个、交通项目18个、商贸项目4个，科技卫生项目8个，旅游项目3个。概算总投资150.46亿元，年内计划完成投资79.92亿元。加强交通设施建设，濮鹤高速公路要在确保质量的前提下，加快工程和配套设施建设进度，年内建成通车；濮信高速公路濮阳段完成路基土方、小型结构物工程；积极做好濮范高速公路的前期准备工作，争取与德商高速公路同时开工；加快汤台铁路改建工程进度，确保年内竣工。国道106线清河头至东明黄河桥段、省道213线吴家庄至西阳邵等7条干线公路和7条县乡公路要开工建设。积极实施黄河滩区道路建设项目。其他方面的重点项目，各责任单位都要集中全力抓紧抓好。同时，今年市政府要办好与全市人民群众生活息息相关的18件实事：（1）建成市下岗失业人员再就业培训基地；（2）开工建设市医疗保险培训康复中心；（3）续建市人才交流中心；（4）完成16万平方米的农村中小学危房改造任务；（5）建成市第一实验小学教学楼；（6）开工建设电视编播大楼；（7）完成市疾病控制中心建设；（8）完成市第五人民医院传染防治病区建设；（9）完成市紧急救援中心建设；（10）开工建设市

119消防指挥中心和城区新增消防栓100处；（11）改造部分城市供水管网；（12）新建供热交换站2座；（13）拓宽建设中原西路、濮上路北段和胜利路西段；（14）完成南海路马颊河桥建设；（15）完成部分背街小巷改造任务；（16）完成部分城区道路和西环游园绿化；（17）提升中心广场、濮上园、世锦园及濮外园景观水平；（18）年内实现“村村通班车”。

围绕提高第三产业的比重，扩大消费需求，加快流通中心建设。要发展大流通，活跃大商贸，改造扩大消费品市场，积极建设专业批发市场，努力培育科技、人才、劳动力等生产要素市场。大力发展连锁经营、物流配送等现代流通方式，扩大城乡消费市场。进一步消除限制消费的各种障碍，完善和落实促进消费的各种政策措施。培育新的消费热点，鼓励居民扩大旅游、通信、教育、体育健身和文化消费。

（八）加强财政金融工作，更好地支持经济社会发展

要依法强化税收征管，做到应收尽收，确保财政收入稳定增长。积极争取中央、省财政转移支付，增加可用财力。要加强支出管理，确保机关事业单位职工工资和离退休人员离退休费按时足额发放，确保农村中小学教师工资按国家现行工资标准按时足额发放，任何地方不得发生拖欠。增加支持解决“三农”问题、国有企业改革、扩大就业、完善社会保障体系等方面的支出。市、县都要建立偿债准备金，提高政府偿债能力。完善国库集中收付和政府采购制度，落实“收支两条线”政策，严格财政监督，确保财政资金规范运行。金融机构要用足用活政策，增加信贷投入，促进地方经济发展。银企之间要加强沟通，实现“双赢”。各级政府要搞好服务，为金融机构创造良好的经营环境。

（九）努力扩大就业和再就业，切实做好社会保障工作

实施积极的就业政策，扩大就业和再就业。各级政府要足额安排再就业资金，落实小额贷款、税费减免等优惠政策，鼓励下岗失业人员自主创业、自谋职业。注重发展劳动密集型产业，吸纳更多的劳动力就业。充分利用企业非主业资产和闲置资产，创造新的就业岗位，安置分流下岗职工。政府投资开发的公益性岗位，优先安排“4050”人员就业。各类企事业单位要积极接收大学毕业生，提高大学生就业率。做好复员转业军人的安置工作，落实鼓励复员转业军人自主创业的各项政策。进一步完善社会保障体系，抓好“两个确保”和城市“低保”工作。确保下岗职工基本生活费和企业离退休人员养老金按时足额发放。把符合条件的城市贫困居民都纳入最低生活保障范围。积极稳妥地推进下岗职工再就业中心向失业保险并轨。以个体、私营和外资企业为重点，扩大城镇养老、医疗、失业等社会保险覆盖面。加大对各类参保企业社会保险费的征缴力度。完善对低收入者的救助制度，解决城乡特殊困难家庭的实际困难。鼓励有条件的地方建立农

村最低生活保障制度。要认真解决建设领域拖欠工程款和农民工工资问题，不准发生新的拖欠。

（十）统筹经济社会协调发展，推动社会主义精神文明建设和民主法制建设

大力实施“科教兴濮”战略。加大科技创新力度，建立和完善企业技术创新机制，抓好科技成果的引进、示范和推广，提高科技成果转化率。坚持教育优先发展的战略地位，落实扶持农村教育的各项政策措施。抓好基础教育，统筹规划城乡中小学布局，巩固提高“普九”成果。加快发展高中阶段教育，积极发展职业教育和成人教育。广泛开展爱国卫生运动，完善公共卫生体系，提高城乡居民健康水平。完善全市疾病预防控制体系、突发公共卫生事件医疗救治体系、重大疫情信息网络体系和卫生执法监督体系，完成五县卫生防疫站扩建工程。抓好农村乡镇中心卫生院建设，深化新型农村合作医疗试点工作。加强基层文化设施建设，开展丰富多彩的文化活动，丰富人民群众的文化生活。搞好文物保护和利用，做好申报国家历史文化名城工作。坚持不懈地开展“扫黄打非”斗争。广泛开展群众性体育活动，增强人民体质，办好市六运会。加强人口与计划生育工作，稳定低生育水平，提高出生人口素质。完善资源有偿使用机制，合理保护和

利用自然资源。加强环境保护，抓好市辖流域水污染防治，综合整治城市环境。做好创建生态示范区工作。以就业、教育、医疗、住房、用电、药品、水7个方面的价格为重点，深化价格改革，维护社会稳定。健全经济运行监测体系，完成全市经济普查任务。继续做好广播电视、人防、外事侨务、民族宗教、气象、史志、档案、地震、残疾人事业等工作。

以邓小平理论和“三个代表”重要思想为指导，深入贯彻党的基本路线、基本纲领、基本经验，广泛宣传党的十六大和十六届三中全会精神。认真组织学习宪法，搞好“四五”普法宣传教育，在全社会营造学法、懂法、守法的良好环境。完善村民自治和城市居民自治，推进基层民主建设。完善职工代表大会制度，保障职工合法权益。做好法律援助工作。认真贯彻公民道德建设实施纲要，弘扬爱国主义精神，加强社会公德、职业道德和家庭美德教育。崇尚科学，反对迷信，倡导文明健康的生活方式。开展“道德规范进万家”、“诚实守信万人行”活动。全民动员，大力开展创建文明城市活动。加强国防教育，巩固军政军民团结，争创全国双拥模范城市。

要落实社会治安综合治理各项措施，推进社会治安防控体系建设。严厉打击黑恶势力和严重暴力犯罪活动。加大对突出治安问题的整治力度，创建省综合治理先进市，保障人民群众安居乐业。深入开展同邪教组织的斗争。要正确处理新形势下人民内部矛盾，健全预防、化解各种矛盾和纠纷的机制，重视做好人民来信来访工作，维护正常社会秩序和社会稳定。

进一步整顿和规范市场经济秩序，优化发展环境。把集中整治与建立长效机制结合起来，继续开展企业经营环境等专项整治工作，坚决制止对企业乱摊派、乱收费、乱罚款、乱检查。深入开展打假打诈联合行动，保护知识产权，严厉打击制售假冒伪劣食品、药品、医疗器械、农资等违法犯罪活动。加强食品安全卫生检查，实施“食品放心工程”。打造诚信濮阳，加强社会信用体系建设，规范行业信用机制，逐步建立企业、中介机构信用档案和个人诚信体系，在全社会营造诚实守信、公平竞争的发展环境。加强安全生产和安全监察，落实安全生产责任制，坚决遏制重特大事故的发生。

四、加强政府自身建设

在全面建设小康社会的进程中，政府工作担负着繁重而艰巨的任务。新形势、新任务要求我们必须坚持实行科学民主决策、坚持依法行政和加强行政监督三项基本准则，进一步加强自身建设，提高工作效率和工作水平，努力建设服务型政府。

推进政府职能转变。把政府管理经济的职能转变到主要为市场主体服务和创造良好发展环境上，更加注重履行社会管理和公共服务职能。深入开展机关效能建设，积极改进行政管理手段，推行电子政务，降低行政成本，提高行政效率。健全政务公开制、服务承诺制、限期办结制、首问负责制、失职追究制等各项制度，办好行政服务中心，加快县（区）、乡（镇）行政服务中心建设步伐，提高行政能力和管理水平。按照省统一部署和要求，完成市县政府机构改革任务。

坚持科学民主决策。建立和完善重大问题集体决策制度、专家咨询制度、社会公示和社会听证制度、决策责任制度。所有重大决策，都要在深入调查研究、广泛听取意见、进行充分论证的基础上，由集体讨论决定。这要作为政府的一项基本工作制度，长期坚持下去。

全面推行依法行政。政府要按照法定权限和程序行使权力、履行职责。所有政府工作人员都要遵守宪法和法律，学会并善于依法处理经济社会事务。加强行政执法监督，促进严格执法、公正执法和文明执法。认真实施《行政许可法》，提高依法行政水平。

自觉接受人民监督。政府的权力是人民赋予的，必须对人民负责，为人民谋利益，接受人民监督。要自觉接受同级人民代表大会及其常委会的监督，接受人民政协的民主监督，认真听取民主党派、工商联、无党派人士和各人民团体的意见，发挥工会、共青团、妇联等人民团体的桥梁纽带作用。同时，接受新闻舆论和社会公众监督；重视人民群众通过行政复议、行政诉讼等法定渠道，对政府机关及其工作人员的监督；加强政府系统内部监督，支持监察、审计部门依法履行监督职责。

加强政风廉政建设和公务员队伍建设。要弘扬求真务实精神，树立正确的政绩观，着力解决改革发展过程中的实际问题，倾力解决群众生活中的紧迫问题和涉及群众切身利益的具体问题，使政府各项工作经得起实践、群众和历史的检验。全体政府工作人员特别是各级领导干部，要牢固树立执政为民的思想，忠于职守，勤奋工作，转变作风，廉洁从政。要以对党和人民高度负责的态度，时刻保持奋发有为的蓬勃朝气、开拓创新的昂扬锐气、清正廉洁的浩然正气，努力开创各项工作的新局面。

各位代表，我们肩负的任务光荣而艰巨。让我们在省委、省政府和市委的领导下，高举邓小平理论伟大旗帜，全面贯彻“三个代表”重要思想，认真落实党的十六大和十六届三中全会精神，抓住机遇，埋头苦干，加快发展，为早日实现全面建设小康社会目标而奋斗！
